# The Impact of Visual Field Loss on Driving Skills: A Systematic Narrative Review

**DOI:** 10.22599/bioj.129

**Published:** 2019-04-16

**Authors:** Gemma Patterson, Claire Howard, Lauren Hepworth, Fiona Rowe

**Affiliations:** 1 NHS Greater Glasgow and Clyde, GB; 2 Salford Royal NHS Foundation Trust, University of Liverpool, GB

**Keywords:** visual field loss, driving, compensation, impact, binocular, monocular

## Abstract

**Purpose:**

To review the evidence on the impact of visual field loss on skills required for driving.

**Methods:**

A literature search was undertaken using a systematic approach. Papers within scope were identified by two independent reviewers, and papers were grouped into similar themes for discussion.

**Key findings:**

Evidence suggests that both binocular and monocular visual field defects have a negative impact on driving skills. Both central and peripheral cause difficulties, but the degree of impact is dependent on the defect severity and compensation ability. Many factors that affect compensation to visual field loss and the effects of visual field loss on driving skills are discussed, including cognitive status, age and duration of visual field loss. In summary, in central visual field loss compensation, strategies include reduction of overall driving speed; whereas, in peripheral field loss, increased scanning is reported to aid adaptation.

**Conclusions:**

For driving, there is evidence that complete and/or binocular visual field loss poses more of a difficulty than partial and/or monocular loss, and central defects cause more problems than peripheral defects. A lack of evidence exists concerning the impact of superior versus inferior defects. The level of peripheral vision loss that is incompatible with safe driving remains unknown, as compensation abilities vary widely between individuals. This review highlights a lack of evidence in relation to the impact of visual field loss on driving skills. Further research is required to strengthen the evidence to allow clinicians to better support people with visual field loss with driving advice.

## Introduction

Visual field loss can affect one eye (monocular) or both eyes (binocular) and may affect the central or peripheral visual field or a combination. Common causes include stroke, glaucoma, diabetic retinopathy and age-related macular degeneration (ARMD). Many of these ocular conditions are age related, and according to the Office of National Statistics, the UK population is ageing; therefore, visual field impairment is projected to increase in the future (Office of National Statistics 2018). Stroke, glaucoma and diabetic retinopathy are generally associated with peripheral field loss and ARMD with central visual field loss. Visual fields can also be affected from a younger age by less-frequent conditions, such as retinitis pigmentosa and Stargardt’s disease.

The sensory information relevant to driving is predominately visual (Sivak 1996). Thus, anything affecting vision has the potential to affect driving ability. Driving is challenging and potentially hazardous for those with visual field loss, because the road is a dynamic environment. The impact of visual field loss on driving will depend upon a combination of factors, such as extent of defect, location and ability to compensate. Important driving components often affected include steering, lane position, traffic-gap judgement, speed, blindside detection and collision avoidance (Alberti et al. 2014, 2009; Bowers et al. 2005; Tant et al. 2002; Wood et al. 2009).

Knowledge of diverse visual field loss and its impact on the various components of driving is paramount in developing rehabilitation options. This study aims to review the evidence on the impact of visual field loss on the skills required for driving. The primary objective is to examine how extent and location of visual field defects affect driving components and a persons’ ability to compensate. The secondary objective is to consider the legal aspects of driving in relation to the legal restrictions for driving with visual field loss.

## Methods

The PRISMA checklist was used throughout the process to assist in adhering to best practices in conducting a systematic review (Moher et al. 2009).

## Search strategy

A systematic search strategy was used to search the following key electronic databases: MEDLINE (1948 to June 2018), SCOPUS (1823 to June 2018), CINAHL (1937 to June 2018) and PsycINFO (1887 to June 2018). Citation tracking was performed using Web of Science cited reference search, and reference lists of included articles were searched manually. Search terms included a variety of MESH terms and alternatives in relation to visual field loss and driving outlined in Table 1.

**Table 1:** Search Terms.


Visual Fields/	Automobile Driving/
Hemianopsia/	Accidents, Traffic/
Scotoma/	driving
visual field loss	on-road
visual field defect	simulation
quadrantanopia	simulator
	hazard detection
	hazard perception
	collision avoidance
	lane position
OR	OR
AND	


## Definitions

Complete homonymous hemianopia is defined as a loss of visual field to one side from central fixation outwards.

Partial homonymous hemianopia is defined as a loss of visual field to one side that is incomplete, with some residual vision on the affected side.

Macular splitting involves the central area of vision (i.e., the area of best visual function at the centre of fixation).

Macular sparing is where a small central area of functioning vision on the side of the loss is preserved.

Compensation means the steps taken by an individual to continue their daily lives without detriment from their visual field loss. There is a lack of evidence as to what these steps involve (Howard and Rowe 2018).

## Inclusion and exclusion criteria

Articles related to visual field loss and driving performance were included. Articles that discussed other visual impairments alongside visual field loss had to discuss visual field loss separately to be included. Studies where interventions were used to enhance driving performance were excluded, along with review articles and single case studies.

## Selection of studies

The titles and abstracts identified were screened by two independent reviewers using the prestated inclusion criteria. Full papers of any studies considered potentially relevant were then considered collectively by the team and grouped into similar themes for discussion.

## Quality assessment

All articles were assessed for methodological quality using the QualSyst tool (Kmet et al. 2004). The scoring system consists of 14 criteria accompanied by detailed instructions to guide decision-making. For each criterion, the article had the potential to be awarded points (yes = 2, partial = 1, no = 0), with a maximum 28 points available in the quantitative version. Points were then converted into a percentage, taking into account criteria that were not applicable depending on the study design. A quality score of >80% is defined as strong, 71–80% is defined as good, 55–70% is considered adequate and <55% is considered limited (Kmet et al. 2004). The score for exclusion was determined to be less than 55%.

## Results

**Figure 1:**
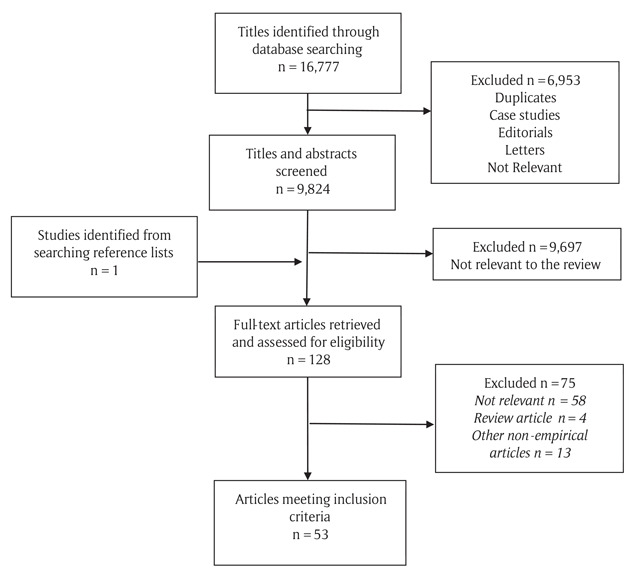
Flowchart of the pathway for inclusion of articles, using a modified PRISMA diagram (Moher et al. 2009).

In total, 53 articles were found to be relevant to this review. Following quality assessment, all articles met the criteria of >55%; therefore, none were excluded. Results of the search are outlined in Figure 1. The articles are discussed in the relevant sections according to the identified themes. The quality of the included articles ranged from good to very strong (70%–100%). The characteristics and quality rating for each included study are outlined in Appendix 1.

## Extent of visual field loss

Conflicting results have been obtained regarding the impact of homonymous visual field loss on driving. The driving deficits reported include inappropriate lane positioning, space judgement, inconsistent steering and increased risk of collisions (Bowers et al. 2009; Kooijman et al. 2004; Kunimatsu-Sanuki et al. 2015; Lövsund et al. 1991; McGwin et al. 2015; Ono et al. 2015; Rubin et al. 2007; Szlyk et al. 1993, 2005; Tant et al. 2002). On the other hand, further studies found little difference in performance between hemianopes and those with full fields (Schulte et al. 1999; Wood et al. 2009). Differences may be due to methodological variations, for example, whether the assessment was on-road or simulated (Wood et al. 2009). Other potential factors are sample size, inclusion criteria and time since onset/adaptation time.

Quadrantanopia is a less extensive visual field defect that affects a quarter of the visual field area. Safe driving appears to be more achievable with this defect than in hemianopia and is likely due to the lesser extent of visual field loss, amongst other factors (Elgin et al. 2010; Parker et al. 2011; Racette and Casson 2005; Wood et al. 2009, 2011). A number of prospective on-road assessment studies used similar inclusion and exclusion criteria for selecting subjects (Elgin et al. 2010; Parker et al. 2011; Wood et al. 2009, 2011). Comparison of on-road performance was made, and 88% (Wood et al. 2009), 87.5% (Elgin et al. 2010) and 87% (Parker et al. 2011) of subjects were found to be safe drivers. Wood et al. (2011) proposed that safe drivers adapted by means of additional head movements towards the affected area, better lane observance and a reduction in abrupt braking. Assessment of compensatory mechanisms, such as saccades and head movements, were not quantified but were subjectively graded (Wood et al. 2009).

A further study involving a simulated driving task found poor compensation among those with quadrantanopia (Lövsund et al. 1991). This study had several limitations, including a small sample size and lack of quantitative analysis, which should be considered. In general, research demonstrates many drivers with quadrantanopia can drive comparatively well, although there were numerous constituent driving actions that were less well executed. These included lane position, gap appraisal and steering smoothness (Elgin et al. 2010; Wood et al. 2009, 2011).

Few studies have considered whether the location of quadrantanopic defects are of significance in driving. Studies that have investigated the impact of whether the defect is inferior or superior have often combined results due to small sample sizes (Elgin et al. 2010; Parker et al. 2011; Wood et al. 2011). Of eight subjects with quadrantanopia, Wood et al. (2009) found the superior field was affected in five instances and the inferior field was affected in three instances. However, the results were not reported individually.

An analysis of requests for exemptions from the visual field standard by Dow (2011) found the location of the defect in both hemianopia and quadrantanopia independent to the outcome of the driving evaluation. They state that, in theory, an intact inferior field is fundamental to safe driving because most of the external action occurs in this area of the visual field. All four subjects with an inferior altitudinal defect passed an on-road practical fitness to drive assessment. In this instance, an inferior defect was not a contraindication to driving, and these individuals were considered by the authors to have sufficiently compensated.

Bilateral altitudinal visual field loss can occur as a result of bilateral lesions of the occipital lobe (Rowe 2016). Although both inferior and superior altitudinal defects can occur as a result of stroke, gaps exist in the literature as to its impact on driving. Bowers et al. (2005) found that restriction of the vertical binocular visual field was significantly related to poorer performance with regards to speed matching when changing lanes, poorer lane positioning when following a curve in the road and worse anticipatory skills. Glen et al. (2014) found that a simulated superior altitudinal defect had more of an impact in a hazard defection task than an inferior defect. This was, however, much removed from an on-road situation because it focused solely on hazard detection without participants controlling a vehicle.

Logic would suggest inferior altitudinal defects would cause difficulty checking side-view mirrors and the speedometer and would cause impaired awareness of what is occurring directly in front of the vehicle. Meanwhile, superior defects could also have a negative impact on driving because upward saccades would be necessary to check the rearview mirror or to read the road conditions and traffic signs/signals ahead and plan sufficiently for approaching situations. The mirror could be fixated with a non-foveal eccentric visual area similar to an individual with any macular defect (Bronstad et al. 2015).

A study simulating concentric constriction of the visual field, associated with increased number of traffic accidents, indicated that a retained central visual field of 10° to 15° may be important for avoiding collisions in places where there is a straight road with a good view (Udagawa et al. 2018).

Limited research has been undertaken specifically focusing on the impact of macular sparing hemianopia on driving ability. In many studies, those with macular sparing have been grouped together with those with complete hemianopia with no distinction made. In some studies where macular sparing hemianopia was outlined initially, specific data for this sub-category was missing from the results (Elgin et al. 2010; Parker et al. 2011; Wood et al. 2009, 2011).

One study found a stronger negative correlation existed between those with hemianopia in whom the central 30° was spared and number of collisions than in those where sparing pertained to another area within the affected hemifield (Papageorgiou et al. 2012). This would indicate that in a virtual driving setting the central field may play an important role in accident prevention. This finding supports the European visual field standard, which stipulates visual field loss cannot not be present within a 20° radius of central fixation for licence holders (Tajani 2009).

Another complete visual field loss is bitemporal hemianopia due to chiasmal pathology. No studies were identified in this review that investigated the impact of this defect on driving ability. However, this gap in the literature could be explained, given that it constitutes an automatic driving disqualification. Due to the existence of post-fixational blindness in bitemporal defects, a driver would struggle with the immediate cone of visual field loss behind the point of fixation (Rowe 1996). Safety would be of major concern as time spent using compensatory head movements to alter position would significantly lessen time spent reading the road conditions ahead.

The consensus reached was that partial visual field loss, such as incomplete hemianopia and quadrantanopia, has less of a negative impact on a person’s driving ability than complete visual field loss. The results from the on-road studies again indicate that some individuals demonstrate safe driving. However, the flaws of the studies also need to be considered. For instance, Elgin et al. (2010) noted that driving rehabilitation specialists made verbal interventions in 50% of the quadrantanopia assessments as opposed to only 16.7% of the control group. This calls into question how many of those rated as safe would be competent if unprompted or unaccompanied.

## Peripheral versus central loss

Peripheral loss can occur gradually with eye conditions, such as diabetic retinopathy or glaucoma, or can be more sudden in onset, such as following a stroke. Hu et al. (2015) reported that field loss in glaucoma is dominated by superior visual field loss, which is associated with a higher incidence of vehicle collisions (Kwon et al. 2016; Tanabe et al. 2011). Gracitelli et al. (2015) and Tanabe et al. (2011) recorded self-reported accident rates from 9.4% to 25%, respectively, in severe cases of glaucoma. Accident rates recorded will vary given the nature of self-reporting, and those with more severe field loss may self-limit the extent to which they drive. It is likely that visual field loss in glaucoma affects visual search performance similarly to that found by Smith et al. (2011). However visual strategy for objects in photographs undoubtedly differs to that undertaken in a dynamic road situation.

Studies have been undertaken that have investigated the impact gradual peripheral visual field loss has on driving ability, and these report longer search times, more fixations with shorter durations and more errors than in individuals without field defects (Coeckelbergh et al. 2002a, 2002b, 2004; Kübler et al. 2015; Szlyk et al. 1993, 1995; Wood and Troutbeck 1992, 1994). Additionally, those with peripheral loss made more lane boundary crossings and were less able to maintain a steady lane position (Coeckelbergh et al. 2002a; Wood and Troutbeck 1992). This may have been due to subjects having to make more head and eye movements to obtain an overview of their surroundings. These findings regarding the variability of lane position are at odds with those of Szlyk et al. (1993, 1995) who found that subjects with central loss were likely to make more lane boundary crossings than those with peripheral loss.

Conversely, longer breaking response times and reaction times in those with peripheral loss have been reported, although these findings are inconsistent (Coeckelbergh et al. 2002b; Szlyk et al. 1993; Wood and Troutbeck 1994). In studies where practical fitness to drive assessments were undertaken, 42% to 50% of those with peripheral loss passed (Coeckelbergh et al. 2002a, 2004; Kübler et al. 2015). Key compensations appear to be a reduction in speed (Wood and Troutbeck 1992, 1994) and increased scanning (Coeckelbergh et al. 2002a; Kübler et al. 2015).

Simulator studies and on-the-road assessments have been undertaken to investigate the driving impact of central visual field loss caused by scotomas in ARMD (Coeckelbergh et al. 2002a, 2002b, 2004; Wood et al. 2018). Deficits recorded were lane boundary crossings, accidents, greater braking response times and motion sensitivity problems compared to those without visual field loss (Coeckelbergh et al. 2002a, 2004; Szlyk et al. 1995; Wood et al. 2018). Coeckelbergh et al. (2002a, 2004) reported reduced response times, despite subjects with central visual field loss driving on average 3km/hour slower than counterparts with peripheral loss. In this study, those with central visual field loss drove at a mean speed of 67 km/hour, compared to 70 km/hour in normally sighted control subjects. These findings are further validated by several studies that found those with central defects display longer search times (Bertera 1988; Henderson et al. 1997; Murphy and Foley-Fisher 1988). Such difficulties are likely due to these individuals trying to obtain as much information from their periphery as they can.

A smaller proportion of drivers with central loss were able to compensate for defects than that previously reported in peripheral loss. In the two on-the-road assessments studies, only 22% and 25%, respectively, of those with central loss passed (Coeckelbergh et al. 2002a, 2004).

Central visual field loss can also result from homonymous scotomas, which occur in stroke as a result of calcarine branch artery occlusion or, rarely, due to the involvement of the macular fibres at the occipital lobe (Petzold and Plant 2005). Paracentral defects are not uncommon, accounting for at least 20% of incomplete homonymous defects (Zhang et al. 2006). Under binocular viewing conditions, a central visual field defect of one eye is usually compensated by the other eye; however, this is not so in cases where the defect is bilateral and homonymous. In such defects, the individual must scan to the affected side to detect objects that could otherwise be missed.

A small study by Bronstad et al. (2011) investigated the ability of three subjects with paracentral, homonymous scotomas to detect pedestrians whilst in a driving simulator. The study found that pedestrians appearing in the affected side were less likely to be detected and reaction times were longer. This group further evaluated the impact of central loss on reaction time to pedestrians in a simulator study in comparison to controls (Bronstad et al. 2013). Their results were consistent with their previous study, indicating greater detection failures in areas of visual field loss: 6.4% compared with 0.2% in controls (Bronstad et al. 2011, 2013). Those with central loss also reacted more slowly to pedestrians in their blind area and missed more responses: 29% versus 3% by controls (Bronstad et al. 2013). This work was updated in 2016 when the effect of central field loss on vehicle control was evaluated (Bronstad et al. 2016). This study highlighted a higher steering wheel reversal rate in drivers with central visual field loss, suggesting that these visually impaired drivers had to allocate extra steering effort to maintain their lane position, which could in turn reduce attentional resources for other driving tasks.

The extent to which bilateral central scotomas compromise driving ability depends upon the defect location and size. Bronstad et al. (2015) proposed that regardless of location, a scotoma could cause delayed responses to hazards, in that gaze movements might occasionally place on-road hazards into the scotoma area and delay detection. In the UK, DVLA guidelines stipulate an individual with significant central loss does not meet the requirements for Group 1 licences; only scattered single missed points or a single cluster of up to three adjoining points on Esterman assessment are considered acceptable (Driving and Vehicle Licensing Agency 2018). In other countries, it may be permissible for individuals to continue driving provided they meet the minimum specified driving requirements of their governments.

In summary, both central and peripheral field loss cause difficulties with regards to driving. The degree to which this occurs depends upon both the extent of the defect and compensation ability.

## Monocular versus binocular field loss

Several studies have suggested that monocularity does not affect driving performance. In most studies, the monocular condition was simulated (Wood and Troutbeck 1992, 1994). No difference was found with regards to steering variables between monocular drivers and age-matched, binocular controls (McKnight et al. 1991). This was due to the visual field loss being compensated for by the field of the fellow eye as the central fields of both eyes overlap (Dow 2011).

On the other hand, the findings of McGwin et al. (2005) contradict this proposal. Patients with moderate to severe visual field loss within the central 24 degree radius in the worse functioning eye were found to be at increased risk of multi-vehicle collision. Kwon et al. (2016) and Tanabe et al. (2011) reported a respective 1.65 times higher incidence of collisions and a statistically significant (*p* = 0.007) association between collisions and severe visual defect in the worse eye but not in the better visual field or worse integrated visual field. Gracitelli et al. (2015) considered collisions in glaucoma and found that those with binocular visual field loss were not at increased risk compared to those with monocular loss. These results confirm that both monocular and binocular visual field loss can be associated with collisions.

## Detection and collision avoidance

Numerous simulator studies address detection and collision avoidance in homonymous hemianopia (Alberti et al. 2014; Bowers et al. 2009; Papageorgiou et al. 2012). All found deficits in blind side detection. These studies consisted of comparable sample sizes and similar criteria: results for hemianopic subjects were compared with matched controls. The studies differ in that two had dynamic obstacle presentation (Alberti et al. 2014; Papageorgiou et al. 2012) creating a more realistic situation, while in the others, objects remained stationary (Bowers et al. 2009). A further study included the use of both stationary and approaching pedestrians in a detection task (Alberti et al. 2014). In this study, drivers with hemianopia exhibited significant blind-side detection deficits. Even when approaching pedestrians were detected, responses were often too late to avoid a potential collision. The purpose of these studies was to evaluate the impact hemianopia has on collision avoidance, albeit in a controlled and simulated environment.

In these studies, miss rates (i.e., the percentage of obstacles participants failed to detect) and pedestrian detection were recorded. Bowers et al. (2009) reported a blind side median miss rate of 60% in hemianopia versus 0% in normally sighted controls. It is important to note these studies were all conducted in conditions when driving on the right side of the road. In a further study, those with left-sided homonymous hemianopia detected 46% of pedestrians compared to only 8% in those with right-sided homonymous hemianopia on extreme left and right gaze, respectively (Bowers et al. 2014). Similarly, Alberti et al. (2014) reported miss rates being significantly higher and reaction times longer in the blind side. However, miss rates were found to be less for approaching targets than stationary ones. In contrast, Bowers et al. (2009) reported variation between performance for left- versus right-sided field loss was not obvious. Another study evaluated collision rate in hemianopic subjects versus controls in a simulator under two traffic densities of ascending difficulty (Papageorgiou et al. 2012). At the 50% density, there was little difference in performance. However, at 75% density, hemianopes significantly averaged two accidents more than controls. The results support Bowers et al. (2009) earlier report that those with homonymous visual field loss experience difficulties under virtual-driving conditions despite the difference in method of moving rather than stationary obstacles. Performance variation between controls and those with homonymous visual field loss was less and may represent superior detection scores on impact evasion tasks with dynamic objects compared to stationary ones (Papageorgiou et al. 2012). These differences may be linked to static-kinetic dissociation (i.e., Riddoch phenomenon), whereby individuals notice objects in motion more readily than stationary ones (Schiller et al. 2006).

Fishman et al. (1981) reported the driving performance of 42 individuals with retinitis pigmentosa causing varying degrees of central and peripheral visual field loss. Overall, affected patients were more likely to be involved in road accidents than normal controls. Lastly, Lee et al. (2016) conducted a study using driving simulator eyeglasses that reduced healthy people’s field of view to approximately 10 degrees. They reported that reducing speed was effective in reducing the risk of collision, compared to looking around frequently.

## Lane position

Lane position was frequently reported as being affected in hemianopia (Szlyk et al. 1993; Tant et al. 2002). A simulator study by Bowers et al. (2010) investigated the position adopted by those with hemianopia. They found that drivers with right visual field loss adopted a lane position significantly left to that of motorists with full visual fields on straight and curved stretches of the road. Drivers with left visual field loss performed similarly to controls but took a more rightward path on left turns (Bowers et al. 2010). These findings are at odds with Tant et al. (2002) who found that about one quarter of subjects with right loss employed a lane position closer to their right boundary. These findings were qualitative in that lane position was not formally quantified.

## Vehicle speed

A further aspect of driving that can be altered by homonymous visual field loss is speed. Bowers et al. (2009) found that, on average, the speed of drivers with hemianopia were less than that of controls. This was significant in both rural (*p* = 0.002) and city (*p* = 0.044) driving environments. Although this relationship was identified, there was no significant correlation between reduction in speed and improved blind side detection rates.

## Compensation for visual field loss

There are numerous compensation strategies for visual field loss (Coeckelbergh et al. 2004). The results of several studies suggest some subjects who fail to meet the legal field requirement for driving can still compensate for their deficit (Bahnemann et al. 2014; Elgin et al. 2010; Hamel et al. 2012; Kasneci et al. 2014; Parker et al. 2011; Racette and Casson 2005; Silveira et al. 2007; Wood et al. 2009).

In hemianopia, Bahnemann et al. (2014) reported that differences in performance could not be accounted for by the side or extent of the defect, but rather, successful performance in tasks appeared to be related to “compensatory mechanisms of visual exploratory behaviour”. Specifically, these consisted of increased saccadic accuracy, increase in the extent of horizontal eye movements and an overall shift of saccades into the blind hemifield. Further to this, Kasneci et al. (2014) highlighted the importance of eye and head movements as a compensatory mechanism. Lee and Itoh (2017) conducted a study whereby constricted visual fields were simulated for driving conditions. Their results indicated that “active head movements are efficient at reducing the number of pedestrian collisions compared with driving without such compensation”. However, these head movements are insufficient in terms of collision avoidance when compared to driving without a visual impairment.

A small but informative study compared the performance of two patients with incomplete right hemianopia with and without compensatory behaviour in a simulator to a healthy control (Hamel et al. 2012). Both compensator and control subjects detected all objects, and no collisions occurred. The compensator was found to perform saccades 1.7 times more frequently than the control, with 63% of saccades covering the affected side.

Alberti et al. (2017) investigated whether individuals with hemianopia were able to spontaneously adapt blind-side scanning in response to differing requirements for detection of pedestrians in a driving simulator. Their results suggested that only a minority of individuals with hemianopia are likely to be able to spontaneously adapt blind-side scanning in response to rapidly changing and unpredictable situations in on-road driving.

A further explorative study found that patients with visual field loss caused by bilateral glaucoma exhibit different eye movements compared to controls when viewing a driving scene (Crabb et al. 2010). On average, patients made more saccades and more fixations than controls to compensate for their impaired vision. It is likely that there are many factors that affect compensation ability. These may include cognitive status, age, duration of visual field loss, reduced speed, scanning, using the lane boundary as a guide and increased head movements.

## Simulator considerations

The advantages of simulator studies are that they are controlled and repeatable. However, they do not reflect real-world stresses that occur whilst driving. From the previously mentioned studies, it is apparent that, under virtual conditions, blind-side detection of pedestrians or vehicles is impaired in hemianopia. It is, however, difficult to ascertain how this correlates with real-world driving. Elgin et al. (2010), in agreement with previous studies, found deficits in steering steadiness and lane position variability (Szlyk et al. 1993; Tant et al. 2002; Wood et al. 2009).

Key differences should be noted between various simulator programmes (Alberti et al. 2014; Bowers et al. 2009, 2010; Papageorgiou et al. 2012). For example, Papageorgiou et al. considered collision rates, whilst Alberti et al. and Bowers et al. avoided setting up collisions. As collisions are generally infrequent in real-world situations, object detection may be a better measure. Furthermore, unlike where the subject has full control of an on-road vehicle, subjects may be unable to stop the vehicle in simulator studies and may use a joy stick rather than a steering wheel (Bowers et al. 2014; Szlyk et al. 1993). This limits the generalisability of the studies’ findings as these conditions are significantly different from real-world driving conditions (Papageorgiou et al. 2012).

## Driving performance

A number of on-road studies have assessed driving, compensatory mechanisms and driver self-reported difficulties (de Haan et al. 2014; Elgin et al. 2010; Kasneci et al. 2014; Parker et al. 2011; Tant et al. 2002). These studies were prospective, with driving being rated by a certified driving rehabilitation specialist or driving instructor, working to the standards set out in government driving tests (de Haan et al. 2014; Elgin et al. 2010; Kasneci et al. 2014; Parker et al. 2011; Tant et al. 2002).

Elgin et al. (2010) recruited 22 subjects with hemianopia; the cause in twelve cases being stroke. They found that these drivers, compared to controls, received a significantly reduced rating for manoeuvres. They also found a significant number of people with hemianopia can drive competently, with 72.7% considered safe to drive on non-interstate and 91.7% on interstate roads (Elgin et al. 2010). Additional issues encountered were that 36.3% had problems adjusting to traffic speed, 40.9% had problems with vehicle control, 27.2% had problems reacting to unexpected events and another 27.2% performed bad manoeuvres (Elgin et al. 2010).

Although those with visual field loss are more likely to have greater difficulty with driving manoeuvres, many studies describe them as safe to drive (Parker et al. 2011; Racette and Casson 2005; Wood et al. 2009). This highlights the place for individual on-road assessment in appropriate cases, as set out by the European Commission’s Directive and implemented in the UK (Driving and Vehicle Licensing Agency 2018; Kasneci et al. 2014; Tajani 2009). An important finding was that drivers with hemianopia who were rated as unsafe were not likely to report greater difficulty driving than those regarded as safe (Parker et al. 2011). The majority of those that failed assessments had a left hemianopia. However, an important consideration is that left hemianopia was an issue when driving on the right-hand side of roads. Further work is required to explore specific issues with right hemianopia when driving on the left-hand side of the road.

In contrast to the above studies showing safe driving performance, two on-road studies had very different findings (Kooijman et al. 2004; Tant et al. 2002). Both found that just 14% of subjects with hemianopia passed on-road driving assessments. These discrepancies could be accounted for by the inclusion criteria; in both studies, patients had been referred due to suspected concerns over driving ability, thus potentially creating bias.

## Limitations of the systematic review

The majority of studies were conducted in countries in which vehicles are driven on the right side of the road. To generalise to countries where vehicles are driven on the left side of the road, adjustments are required in terms of visual field loss laterality.

The tool used for quality assessment (QualSyst) could be open to subjective interpretation. The use of summary scores to quantify studies could introduce a level of bias into the systematic review (Kmet et al. 2004). The authors used the detailed instructions to reduce this level of bias and subjective variation.

## Conclusions

In summary, visual field loss has a negative impact on the skills required for driving, and drivers use a number of strategies to compensate for this. This review of available literature highlights a lack of evidence in relation to the impact of visual field loss on driving skills. Without this evidence, clinicians are unable to fully support people with visual field loss with driving advice and recommendations. This, in turn, limits the impact of any driving rehabilitation offered. An important future research question to consider is how best to assess if someone with visual field loss has compensated sufficiently to drive safely or is no longer safe to drive. Often, a driving assessment is the only way to fully understand the impact of visual impairment on driving ability, which is time consuming, has cost implications and can be a stressful experience for the person involved.

This review does show that complete visual field loss poses more of a difficulty than partial loss, central defects cause more problems than peripheral and a lack of evidence exists concerning the impact of superior versus inferior defects.

Whilst most studies found that visual field loss impacts driving performance, the level of loss that is incompatible with safe driving remains uncertain. This review outlines several compensatory mechanisms that help such individuals improve their driving safety. In central visual field loss compensation, strategies include reduction of overall driving speed; whereas, in peripheral field loss, increased scanning is reported to aid adaptation. Within this review, it is not reported how development of such compensations can be aided. What is clear is that a period of time must elapse in order for individuals to develop compensatory strategies to adapt to visual field loss, particularly when the visual impairment is of sudden onset. Given this, individual driving skill assessments are recommended, rather than comprehensive prohibitions.

## Additional File

The additional file for this article can be found as follows:

10.22599/bioj.129.s1 Appendix 1. Characteristic of included studies and quality rating.
