# Supplementary material for: The Impact of Visual Field Loss on Driving Skills: A Systematic Narrative Review
Source: Br Ir Orthopt J. 2019 Apr 16;15(1):53–63. doi: 10.22599/bioj.129 (PMC7510550; doi:10.22599/bioj.129)
Supplement: Appendix 1. — Characteristic of included studies and quality rating. [file bioj-15-1-129-s1.pdf]

Appendix 1: Characteristic of included studies and quality rating

ARMD: age-related macular degeneration, RP: retinitis pigmentosa

Study	Design	Ophthalmic condition	Sample size (n=)	On-road or simulation	Key findings	Quality rating (%)
Alberti et al., 2017	Prospective observation	Homonymous hemianopia	12	Simulation	Only a small proportion were able to spontaneously adapt scanning technique to situation without training	Strong 95
Alberti et al., 2014	Prospective observation	Homonymous hemianopia	12	Simulation	Significant hemianopic side detection deficits were found, with the delay often too late to avoid a collision	Strong 95
Bahnemann et al., 2015	Prospective observation	Homonymous hemianopia	14 + 14 matched controls	Simulation	Some with homonymous hemianopia are able to detect unexpected, moving obstacles during simulated driving. Side or extent of the field defect do not explain the differences. Instead to compensatory behaviour	Strong 90
Bertera, 1988	Prospective observation	Scotoma (simulated)	23 healthy participants with simulated scotoma	-	Search time doubled with a 20 min arc simulated central visual field scotoma and eye fixation duration increased by around 15%.	Good 80
Bowers et al., 2014	Prospective observation	Homonymous hemianopia	14	Simulation	Provides a detailed quantification of head scanning behaviour at junctions. Compensation strategies included increased frequency of head scanning to affected side but not in size of head scan. Failure to scan adequately increased the failure of detection of pedestrians at junctions.	Strong 95
Bowers et al., 2010	Prospective observation	Homonymous hemianopia	12 + 12 matched	Simulation	Participants took a lane position which increased the safety margin on their	Strong 91

Study	Design	Ophthalmic condition	Sample size (n=)	On-road or simulation	Key findings	Quality rating (%)
			controls		hemianopic side	
Bowers et al., 2009	Prospective observation	Homonymous hemianopia	12<br>+ 12 matched controls	Simulation	Most participants had blind-side detection rates that may be deemed incompatible with safe driving. Highlighting importance of individual assessments including hazard detection.	Strong<br>86
Bowers et al., 2005	Prospective observation	Moderate visual field loss	28	On-road	Mild to moderate peripheral visual field loss adversely affects specific driving skills in manoeuvres for which a wide field of vision is likely to be important e.g. changing lanes and lane positioning during a curve.	Strong<br>85
Bronstad et al., 2016	Prospective observation	Binocular central visual field loss	19<br>+ 15 controls	Simulation	Most measures of vehicle control similar, those with central field loss had a higher steering wheel reversal rate. Authors concluded more steering effort required to maintain lane position.	Strong<br>86
Bronstad et al., 2015	Prospective observation	Central visual field loss	7<br>+7 matched controls	Simulation	Central field loss delayed detection resulting in being unable to stop for 21% of pedestrians compared to 3% in matched controls.	Strong<br>91
Bronstad et al., 2013	Prospective observation	Central visual field loss	11<br>+ 11 controls	Simulation	Participants with binocular scotoma had delayed reactions to potential hazards in scotoma locations.	Strong<br>86
Coeckelbergh et al., 2004	Prospective observation	Visual field loss: e.g. ARMD, RP, glaucoma	100	On-road	A higher percentage of participants with peripheral visual field loss were able to pass the on-road driving assessment than those with central visual field loss.	Strong<br>90

Study	Design	Ophthalmic condition	Sample size (n=)	On-road or simulation	Key findings	Quality rating (%)
Coeckelbergh et al., 2002a	Prospective observation	Visual field loss: e.g. ARMD, RP, glaucoma	87	Simulation	Participants with central visual field loss compensated by reducing their speed. Participants with peripheral visual field loss demonstrated increased swaying which may be the result of compensatory head/eye movements.	Strong 90
Coeckelbergh et al., 2002b	Prospective observation	Visual field loss: e.g. ARMD, RP, glaucoma	50	On-road	None of the eye movement parameters were significantly related to viewing behaviour during the on-road driving test. Therefore limiting the predictive value of these parameters for performing complex tasks such as driving.	Strong 95
Crabb et al., 2010	Prospective observation	Primary open angle glaucoma	9<br>+ matched controls	Simulation	Participants with bilateral glaucoma exhibited different eye movement behaviour i.e. more saccades and more fixations, compared to visually healthy controls when viewing a driving scene.	Strong 91
de Haan et al., 2014	Prospective observation	Homonymous hemianopia	26	On-road	Visual behaviour was found to be affected in a substantial part of participants, but also tactical and operational aspects. A recommendation was made that training programs aiming to improve practical fitness to drive in individuals with homonymous hemianopia should include compensatory visual scanning and driving aspects such as steering	Strong 90

Study	Design	Ophthalmic condition	Sample size (n=)	On-road or simulation	Key findings	Quality rating (%)
					stability, speed adaptation, and anticipating environmental changes.	
Dow 2011	Retrospective record review	Visual field loss	103	On-road	This study demonstrated that many drivers disqualified due to visual field loss can drive safely. No criteria was identified to permit an accurate prediction of failure of the road test.	Strong 89
Elgin 2010	Prospective observation	Hemianopia/ Quadrantanopia	30 + 30 controls	On-road	Participants with hemianopia and quadrantanopia displayed acceptable driving skills, indistinguishable from controls with no field loss. Highlights the importance of driving assessment.	Strong 91
Fishman et al., 1981	Prospective observation	Retinitis pigmentosa	42 + 87 controls	On-road	Differences in the number of accidents between individuals with RP and controls. No significant correlations were found between central or peripheral field efficiency and number of road accidents.	Strong 82
Glen et al., 2015	Prospective observation	Inferior or superior visual field defects (simulated)	30	Simulation	Simulated defects impaired ability to detect driving hazards compared to performance without a visual field defect. Superior defects had more impact than inferior defects.	Strong 85
Graciteli 2015	Prospective observation	Glaucoma	117	Simulation	Longitudinal driving simulator metrics and useful field loss view divided attention test were independent predictors of collisions.	Strong 91
Hamel et al., 2012	Prospective observation	Homonymous hemianopia	2 + 1 control	Simulation	It would be appropriate to teach compensatory behaviour in a simulated	Strong 83

Study	Design	Ophthalmic condition	Sample size (n=)	On-road or simulation	Key findings	Quality rating (%)
					environment and expose patients to a real driving situation as a second step.	
Haymes et al., 2008	Prospective observation	Glaucoma	20<br>+ 20 controls	On-road	Individuals with glaucoma and slight to moderate visual field loss perform standard driving manoeuvres safely. Although individuals with worse visual field loss had more difficulty with peripheral obstacle/hazard detection and unexpected events.	Strong<br>91
Kasneci et al., 2014	Prospective observation	Post-chiasmal visual field loss and glaucoma	20<br>+ 20 controls	On-road	The extent of visual field loss alone is unable to predict fitness to drive. Compensation to the visual field loss can occur in the form of scanning. Individuals who passed the driving assessment had different exploration patterns than those who failed, with more scanning to the side of the visual field loss with more head and shoulder movements.	Strong<br>86
Kooijman et al., 2004	Prospective observation	Retinal pathology and post chiasmal damage	128	On-road	13-62% passed the on-road test before training. After training 15-45% passed. Training of compensatory viewing improved performance in the on-road test.	Strong<br>85
Kubler et al., 2015	Prospective observation	Glaucoma	6	Simulator	Safe driving behaviour is possible with binocular glaucomatous visual field loss in a virtual reality environment. With a compensatory strategy of increasing visual scanning.	Good<br>77
Kunimatsu et al.,	Case control	Glaucoma	36	Simulator	Patients with advanced glaucoma were	Strong

Study	Design	Ophthalmic condition	Sample size (n=)	On-road or simulation	Key findings	Quality rating (%)
2015	study		+ 36 controls		involved in a significantly higher number of collisions than the matched normal subjects. Simulators may have future potential in educating patients.	86
Kwon et al., 2016	Retrospective population-based study	Glaucoma	2000	On-road	Older drivers with glaucoma were shown to be more likely to have more collisions than those without glaucoma. Visual field loss appeared to contribute to this rather reduced visual acuity or contrast sensitivity.	Strong 95
Lee et al., 2017	Prospective observation	Constricted visual field loss (simulated)	18	Simulation	Compensatory head movements in drivers with visual field loss allowed reaction to pedestrians to be more rapid compared driving while keeping their head fixed. However this compensation mechanism only improves reaction to a certain level.	Strong 85
Lee et al., 2016	Prospective observation	Constricted visual field loss (simulated)	18	Simulation	Reducing speed was effective in reducing the risk of collision compared to looking around frequently.	Good 80
Lovsund et al., 1991	Prospective observation	Visual field loss	31 + 20 controls	Simulation	Most subjects with visual field defects showed an impaired detection capacity for stimuli in the affected parts of the visual field. This indicates that most subjects with homonymous defects cannot compensate for their defect.	Good 73
McGwin et al., 2015	Retrospective cohort	Glaucoma	438	On-road	Individuals with glaucoma and severe visual field loss in the binocular field had a higher rate of at-fault collisions	Strong 100

Study	Design	Ophthalmic condition	Sample size (n=)	On-road or simulation	Key findings	Quality rating (%)
					when compared to those with less impaired or unimpaired binocular visual fields.	
McGwin et al., 2005	Prospective observation	Glaucoma	120<br>+ 120 controls	On-road	Individuals with glaucoma and moderate to severe visual field loss within the central 24° radius of the worse-functioning eye are at an increased risk of involvement in a collision.	Strong<br>100
McKnight et al., 1991	Prospective observation	Monocularity	80	On-road	Monocular drivers were found to have some significant reductions in selected visual capabilities and in certain driving functions compared with binocular drivers. No significant differences were found in safety of most day-to-day driving functions between monocular and binocular drivers.	Strong<br>86
Ono 2015	Prospective observation	Glaucoma	199<br>+ 187 controls	On-road	A significant association between the severity of visual field loss in the worse eye and collisions was found.	Strong<br>95
Papageorgiou et al., 2012	Prospective observation	Homonymous visual field loss	30<br>+ 30 controls	Simulation	The extent of visual field loss was weakly associated with performance in the collision avoidance task. A wide between-subject variation was noted with some with visual field loss performing similarly to controls due to compensation strategies. Therefore individualised assessment of compensatory functional behaviour is required.	Strong<br>86

Study	Design	Ophthalmic condition	Sample size (n=)	On-road or simulation	Key findings	Quality rating (%)
Parker et al., 2011	Prospective observation	Hemianopia and quadrantanopia	24<br>+ 24 matched controls	On-road	Individuals with hemianopic/ quadrantanopic visual field loss on average report more difficulty in driving scenarios that critically rely on peripheral vision for example, lane-keeping and steering steadiness. Not all participants with visual field loss had insight into their on-road driving performance abilities.	Strong<br>91
Racette and Casson, 2005	Retrospective case review	Homonymous visual field loss	131	On-road	Some individuals with visual field loss demonstrated safe driving. The extent of visual field loss was identified as being related to driving fitness but no conclusion could be drawn with regard to the location of the deficit. The need for individualised on-road assessments was highlighted.	Good<br>73
Rubin et al., 2007	Prospective observation	None – crash involvement only	120	-	Glare sensitivity, visual field loss, and UFOV were significant predictors of crash involvement. Acuity, contrast sensitivity, and stereoacuity were not associated with crashes.<br>These results suggest that current vision screening for drivers, based primarily on visual acuity, may miss important<br>aspects of visual impairment.	Strong<br>100
Schulte 1999	Prospective observation	Cerebral visual field loss	9<br>+ 10 controls	Simulation	Findings showed no reliable difference in the performance of visually impaired and normally sighted subjects on a	Strong<br>85

Study	Design	Ophthalmic condition	Sample size (n=)	On-road or simulation	Key findings	Quality rating (%)
					driving simulator. Results indicate that suspension of driving privileges for people having visual field impairments may be unwarranted on the basis of visual field loss alone.	
Silveira et al., 2007	Prospective observation	None	100	On-road	Visual field test results did not predict driving performance accurately for both participants with and without visual field loss. Results bring into question the current Austroads visual field standards for safe driving.	Strong 83
Szlyk et al., 2005	Prospective observation	Glaucoma	40 +17 controls	Simulation	Visual field reduced to less than 100 degrees of horizontal extent may place patients with peripheral field loss at greater accident risk. A higher incidence of real-world and simulator accidents was found for the group with glaucoma.	Strong 91
Szlyk et al., 1995	Prospective observation	ARMD	10 + 11 controls	On-road and simulation	Vision, simulator, and on-road test variables combined with subjective risk taking predicted self-reported real-world accidents in a logistic regression analysis. However, risk taking, rather than simulator or road-test performance, was the most significant predictor for both patients with ARMD and the control group.	Strong 82
Szlyk et al., 1993	Prospective	Hemianopia and	6	Simulation	Age related effects combined with the	Good

Study	Design	Ophthalmic condition	Sample size (n=)	On-road or simulation	Key findings	Quality rating (%)
	observation	quadrantanopia	+ 7 controls		effects of visual field losses in older patients with cerebrovascular accidents had a negative impact on driving skills.	80
Szlyk et al., 1992	Prospective observation	RP	21<br>+ 31 controls	Simulation	Results indicate that visual field loss is a primary correlate of automotive accidents in individuals with RP	Good<br>73
Tanabe et al., 2011	Retrospective cohort	Glaucoma	121<br>+ 144 controls	-	Advanced primary open angle glaucoma with marked visual field defects<br>may be a risk factor for motor vehicle collisions.	Strong<br>100
Tant et al., 2002	Prospective observation	Homonymous hemianopia	28	On-road	A minority of the hemianopic patients passed the driving test. This confirmed that hemianopia should not be an absolute contraindication for practical fitness to drive.	Good<br>80
Udagawa et al., 2018	Prospective observation	Constricted visual field loss (simulated)	88	Simulation	Concentric constriction of the visual field was associated with increased number of traffic accidents. The simulation findings indicated that a visual field of 10˚ to 15˚ may be important for avoiding collisions in places where there is a straight road with a good view.	Strong<br>82
Wood et al., 2018	Prospective observation	ARMD	33<br>+ 50 controls	On-road	Drivers with ARMD can exhibit impairments in their driving performance, particularly during complex driving situations; motion sensitivity was most strongly	Strong<br>95

Study	Design	Ophthalmic condition	Sample size (n=)	On-road or simulation	Key findings	Quality rating (%)
					associated with driving performance.	
Wood et al., 2011	Prospective observation	Hemianopia and quadrantanopia	30<br>+ 30 controls	On-road	Persons with hemianopic and quadrantanopic defects rated as safe to drive compensated by making more head movements into their blind field, combined with more stable lane keeping and less sudden braking.<br>Future research should evaluate whether these characteristics could be trained in rehabilitation programs aimed at improving driving safety in this population.	Strong<br>86
Wood et al., 2009	Prospective observation	Hemianopia and quadrantanopia	30<br>+ 30 controls	On-road	Some drivers with hemianopia or quadrantanopia are fit to drive compared with age-matched control drivers.<br>Results call into question the fairness of governmental policies that categorically deny licensure to persons with hemianopia or quadrantanopia without the opportunity for on-road evaluation.	Strong<br>95
Wood and Troutbeck, 1994	Prospective observation	Cataracts, visual field loss and monocularity (simulated)	14	On-road	Simulated cataract resulted in the greatest detriment to driving performance, followed by binocular visual field restriction. The monocular condition did not significantly affect driving performance for any of the driving tasks assessed.	Strong<br>85
Wood and	Prospective	Constricted	9	On-road	Constriction of the binocular visual field	Adequate

Study	Design	Ophthalmic condition	Sample size (n=)	On-road or simulation	Key findings	Quality rating (%)
Troutbeck, 1992	observation	visual field loss (simulated)			to 40 degrees or less, significantly increased time taken to complete the course, reduced the ability to detect and correctly identify road signs, avoid obstacles and to manoeuvre through limited spaces. Accuracy of road positioning and reversing were also impaired. Constriction of the binocular visual field did not significantly affect speed estimation, stopping distance, or the time taken for the reversing and manoeuvring tasks.	70
